# The Imperfect Sight of the Normal Eye

**Published:** 1918-02

**Authors:** William H. Bates

**Affiliations:** New York


### Journal-Record of Medicine

Successor to Atlanta Medical and Surgical Journal, Established 1855
and Southern Medical Record, Established 1870

**OWNED BY THE ATLANTA MEDICAL JOURNAL COMPANY**

PUBLISHED MONTHLY

*Official Organ Fulton County Medical Society, State Examing Board, Atlanta, Birmingham and Atlantic Railroad Surgeon's Association, Chattahoochee Valley Medical and Surgical Association, Etc.*

A. W. STIRLING, M. D., C. M., D. P. H.; J. S. HURT, B. Ph., M.D.
GEO. M. NILES, M. D., W. J. LOVE, M. D., (Ala.); Associate Editors

E. W. ALLEN, Business Manager

**COLLABORATORS**

W. F. WESTMORELAND, M. D., General Surgery
F. W. McRAE, M. D., Abdominal Surgery
ALLEN H. BUNCE, Medical Societies
E. B. BLOCK, M. D., Diseases of the Nervous System
MICHAEL HOKE, M. D., Orthopedic Surgery
CYRUS W. STRICKLER, M. D., Legal Medicine and Medical Legislation
E. C. DAVIS, A. B., M. D., Obstetrics
E. G. JONES, A. B., M. D., Gynecology
ALLEN H. BUNCE, M. D., Pathology and Bacteriology
R. T. DORSEY, JR., B. S., M. D., Medicine
L. M. GAINES, A. B., M. D., Internal Medicine
GEO. C. MIZELL, M. D., Diseases of the Stomach and Intestines
L. B. CLARKE, M. D., Pediatrics
EDGAR PAULLIN, M. D., Opsonic Medicine
THEODORE TOEPEL, M. D., Mechano Therapy
E. G. BALLENGER, M. D., Diseases of the Genito-Urinary Organs

VOL. LXIV. ATLANTA, GA., FEB., 1918. NO. 11

### THE IMPERFECT SIGHT OF THE NORMAL EYE.

By William H. Bates, M.D., New York.

### Occurrence.

It is generally believed that the normal eye has perfect sight all the time. It has been compared to a perfect machine which is always in good working order. We have been taught that the normal eye is always normal and that the sight is always perfect, no matter what the object regarded may be, whether new, strange, or familiar, whether the light is good or imperfect, or whether the surroundings are pleasant or disagreeable. Even under conditions of nerve strain or bodily disease, the normal eye is expected to have perfect sight always.

A scientific study of the facts has convinced me that this impression so generally believed and taken for granted is far from the truth. After thirty years special study of the refraction of the eye under different conditions I am convinced that the normal eye has imperfect sight most of the time. It is unusual to find persons who can maintain perfect sight continuously longer than a few minutes under the most favorable conditions. Of 20,000 school children studied by me, more than half had normal eyes with perfect sight. Not one of them had perfect sight in each eye every day. The sight of many of them might be good in the morning and imperfect in the afternoon, while many with imperfect sight in the morning would have frequently perfect sight in the afternoon. Many children with normal eyes would read on Snellen test card with perfect sight, a second and different one with imperfect sight. Many children could read some letters of the alphabet with perfect sight but were unable to distinguish other letters of the same size under similar conditions. The amount or the degree of the imperfect sight varied within wide limits from one third of the normal to one tenth or less. The duration of the imperfect sight of the normal eye was also variable. Under some conditions in the classroom the imperfect sight might continue for only a few minutes or less. Under other conditions, however, a small number of pupils, sometimes all the pupils with normal eyes would have sufficient loss of sight to prevent them from seeing writing on the blackboard for days, weeks, or longer.

Adults with normal eyes do not have perfect sight all the time and what has been said of the normal eyes of school children is also true of them. Age is no exception. Persons over seventy years of age with normal eyes have had attacks of loss of sight variable in degree and duration. The reinoscope always indicated an error of refraction when the sight of the normal eye was imperfect. A man eighty years old with normal eyes and perfect sight had periods of imperfect sight which would last from a few minutes to several hours or longer. Retinoscopy always indicated myopia, sometimes 4D or more. Many adults with normal eyes, as well as children, have attacks of color blindness. One patient with normal eyes with perfect sight and perfect color perception in the daytime has always been color blind at night. He had no perception of colors after sunset. It is true that all persons with normal eyes are always less able to distinguish colors correctly during the time that their sight was imperfect. Accidents on railroads, collisions, and other accidents at sea, trolley car accidents, automobiles with their high daily death list, occur usually because the normal eyes of the responsible persons for a time had imperfect sight. Accidents accur when nervous children or adults cross the street. They become confused, blinded, and are struck by automobiles, trolley cars, are injured because, although they had normal eyes, their sight was lost.

**Cause.**

There is but one cause of functional imperfect sight, a strain or effort to see. The normal eye with good sight is at rest, but, with imperfect sight, the reinoscope always indicates an error of refraction sufficient to account for the defect in the vision. The strain may be an unconscious strain or it may produce results on the eyes, pain, discomfort, fatigue, of which the individual may be conscious. Quite often the strain may be a conscious effort without the production of discomfort. In all cases of strain it can be demonstrated that the eyes do not see best the point fixed but some other point to one side—the eyes do not see best where they are looking. If one letter or one word of a line is regarded, other letters or words on the same or other lines will be seen as well or better when the eyes are straining or making an effort to see.

The normal eye can be made to strain consciously by making an effort to see a letter or word better than the one regarded. The vision is always lowered for the letter or word regarded and an error of refraction is always produced. Unfamiliar objects cause eye strain and are never seen perfectly. School children with normal eyes who can read with normal sight small letters one quarter of an inch high at ten feet always have trouble in reading strange writing on the blackboard although the letters may be two inches high. Myopia, temporary or permanent, is always produced. Strange maps always produce imperfect sight in the normal eye because they cause a strain or effort to see.

When the eyes are used for near work the normal eye is seldom properly focussed. The retinoscope has always demonstrated that when an effort or strain is made to see more clearly at twelve inches, twenty inches, or less than twelve inches, the eyes are always focussed at a greater distance with the production of astigmatism, usually temporary, but which has been observed to become permanent. Of course with the eyes not properly focussed the vision is defective. School children and adults learning to read, write, draw, sew, or to do technical work suffer from defective vision although they have normal eyes. This matter is of such practical importance that the attention of teachers should be called to the facts. Many children lose interest in their school work, become truants, incorrigibles, and chronic sufferers from headaches and other neuroses who might have been relieved by proper treatment. I have described the relief obtained by school children when the teachers understood the problem (1).

Light has a very important effect on the vision of the normal eye. An nuexpected strong light always produced defective vision. The vision of all persons is imperfect when the eyes are first exposed to the strong light of the sun or to strong artificial light. Rapid or sudden changes in the intensity of the light always produce defective vision, not always sufficiently great to be manifest to the individual but which can always be demonstrated by careful tests of the vision and by use of the retinoscope. The defective vision produced by strong light may be temporary but it has been observed to continue in many cases for a number of weeks or months. It is never a permanent disability. Persistence in regarding a strong light after a time become a benefit. Some persons have become able to look directly at the strong light of the sun without any loss of vision whatever. When the light is dim or at night, the vision of the normal eye is usually good; but, when an effort is made to see, the vision becomes imperfect and the retinoscope indicates always an error in refraction.

Noise is a frequent cause of defective vision of the normal eye. All persons see imperfectly when they hear an unexpected loud sound. Familiar noises do not lower the vision usually, but unfamiliar, new, or strange noises always do with the production of a temporary error of refraction. Country children from quiet schools, after moving to a noisy city, may suffer from the effects of defective vision for long periods of time, weeks, months. In the classroom they do not do well in their work because their sight is impaired. It is a gross injustice for teachers and others to criticize, scold, punish, or humiliate them.

Moving pictures usually produce defective vision which is always temporary. Some of my patients have complained that they always suffered with pain and had poor sight whenever they regarded the screen with its flickering light. I believe that some years ago when photography was less perfect than it is now the pictures produced a great deal of eye strain, much greater then than at the present time. I always advised my patients under treatment for the cure of defective vision without glasses, to go to the movies frequently, practice central fixation (2), and become accustomed to the flickering light. They soon became able to stand the strain without loss or impairment of their vision. Other lights and reflections from smooth surfaces became less annoying and it seemed true that after the movies were unable to produce a relapse other lights were unable to lower the vision after they were relieved of errors of refraction, myopia, hypermetropia, and astigmatism by treatment.

**Treatment.**

Eye training with the aid of a Snellen test card at ten feet or farther is very successful in correcting and in preventing the imperfect sight of the normal eye. One may use a distant calendar, a sign with small letters, or one small letter far daily practice. The normal eye is readily trained to read the Snellen test card with normal vision or to see other letters or figures or one known small letter at a distance of ten feet or farther. The vision always improves and becomes better than that of the average normal eye. The practice of reading known or familiar letters once daily or more frequently with normal sight by the normal eye is a decided benefit and lessens the tendency to strain when regarding unfamiliar letters of objects.

**Results.**

On my recommendation more than 20,000 school children have practices eye training with the aid of the Snellen test card. The results were of great practical importance. The vision of the normal eye was always improved when the teachers used the method properly. Because the sight was always improved, myopia was always prevented. This is the first published method for the prevention of myopia which was successful. Many children wearing glasses to benefit imperfect sight, pain and fatigue of the eyes, and headaches were relieved so completely that they became able to discard their glasses and obtained more perfect sight and a greater relief to their eye pain and headaches. The eye training demonstrated by the good results obtained that many thousands of children in the schools are wearing glasses that they do not need because their eyes are normal.

Artists, bookeepers, lawyers, physicians, writers, mechanics, and other found their efficiency increased many times with the aid of eye training. Many recruits for the army and navy were found to have imperfect sight and were rejected, although their eyes were normal. Eye training improved their sight. Later they read the Snellen card with perfect sight and were accepted.

**Conclusions.**

1. All persons with normal eyes and perfect sight do not have nromal eyes and perfect sight continuously.

2. The cause is always an effort or strain to see.

3. Treatment by eye training is successful when distant, familiar letters, are read a few moments at least every day.

4. The good results obtained justify the use of the method in all schools, the army, the navy, the merchant marine, on all railroads, in short by everybody who desires or needs continuous perfect sight.—New York Medical Journal.

